# Frequency and mortality of sepsis and septic shock in China: a systematic review and meta-analysis

**DOI:** 10.1186/s12879-022-07543-8

**Published:** 2022-06-21

**Authors:** Yan-Cun Liu, Ying Yao, Mu-Ming Yu, Yu-Lei Gao, An-Long Qi, Tian-Yu Jiang, Zhen-Sen Chen, Song-Tao Shou, Yan-Fen Chai

**Affiliations:** Department of Emergency Medicine, Tianjin Medical University General Hospital, 154 Anshan Road, Tianjin, 300052 China

**Keywords:** Sepsis, Frequency, Mortality, China, Meta-analysis

## Abstract

**Background:**

Sepsis, a life-threatening organ dysfunction induced by infection, is a major public health problem. This study aimed to evaluate the frequency and mortality of sepsis, severe sepsis, and septic shock in China.

**Methods:**

We Searched MEDLINE, Embase, PubMed, and Cochrane Library from 1 January 1992 to 1 June 2020 for studies that reported on the frequency and mortality of sepsis, severe sepsis, and septic shock conducted in China. Random effects models were performed to estimate the pooled frequency and mortality of sepsis, severe sepsis, and septic shock.

**Results:**

Our search yielded 846 results, of which 29 studies were included in this review. The pooled frequency of sepsis was estimated at 33.6% (95% CI 25.9% to 41.3%, I^2^ = 99.2%; p < 0.001), and the pooled mortality of sepsis, severe sepsis and septic shock were 29.0% (95% CI 25.3%–32.8%, I^2^ = 92.1%; p = 0), 31.1% (95% CI 25.3% to 36.9%, I^2^ = 85.8%; p < 0.001) and 37.3% (95% CI 28.6%–46.0%, I^2^ = 93.5%; p < 0.001). There was significant heterogeneity between studies. With a small number of included studies and the changing definition of sepsis, trends in sepsis frequency and mortality were not sufficient for analysis. Epidemiological data on sepsis in the emergency department (ED) are severely lacking, and more research is urgently needed in this area is urgently needed**.**

**Conclusions:**

Our findings indicated that the frequency and mortality of sepsis and septic shock in China were much higher than North America and Europe countries. Based on our results, an extremely high incidence and mortality of sepsis and septic shock in China's mainland requires more healthcare budget support. Epidemiological data on sepsis and septic shock in ED are severely lacking, and more research is urgently needed in this area.

*Trial registration* This systematic review was conducted according to the statement of the preferred reporting items for systematic review (PROSPERO CRD42021243325) and the meta-analysis protocols (PRISMA-P).

**Supplementary Information:**

The online version contains supplementary material available at 10.1186/s12879-022-07543-8.

## Background

Sepsis is a life-threatening immune disorder and organ dysfunction induced by infection and a global health problem [1]. With the evolution of the definition of sepsis from Sepsis-1 to Sepsis-3, the concept of sepsis is more inclined to organ dysfunction. There were 48.9 million incident cases of sepsis worldwide and 11.0 million deaths related to sepsis were estimated, representing 19.7% of all global deaths [2]. However, the frequency and mortality of sepsis vary greatly in different studies. These variations may be explained by variations in the design of the study, the number of centers, the location of patients, geographical region, and the evolving definition of sepsis [1, 3–5].

Recent meta-analysis reviewed the frequency and mortality of sepsis and septic shock in Europe, North America, and Australia [6, 7]. However, with the difference in the healthcare system in China and other countries in Asia, research in countries in Asia was excluded from these systematic reviews. China is the most populous developing country in the world, and at the same time, critical care medicine in China has developed rapidly in the past ten years. Therefore, studying the frequency and mortality of sepsis and septic shock in China will be of great significance in assessing the global situation of the sepsis epidemic In recent years, several high-quality epidemiological studies [8–11] and reviews [12] on sepsis in China have been published. However, due to differences in the included population, diagnostic criteria for sepsis, and study endpoints, the conclusions of the studies were not the same. A systematic review and meta-analysis are urgently needed to accurately reflect the frequency and mortality of sepsis in China. Consequently, we investigated the epidemic trends of sepsis and septic shock from 1992 to 2020, including prospective and retrospective studies to get a clear understanding of the frequency and mortality of sepsis and septic shock in China.

## Methods

This systematic review was conducted according to the statement of the preferred reporting items for systematic review (PROSPERO CRD42021243325) and the meta-analysis protocols (PRISMA-P).

### Search strategy

MEDLINE, the Chinese Biomedical Literature Database, the Chinese Medical Current Content, Embase, PubMed and the Cochrane Library were searched, and we limited our search to publications published between 1 January 1992 and 1 June 2020. We only included studies published in English. We used a comprehensive list of search terms for each database. We use this list in the title of publications: (sepsis OR septic) AND (epidem*, frequen*, prevalence, incidence, OR mortality) AND (China). We also screened existing systematic reviews and checked the reference lists of eligible studies.

### Study selection criteria

Based on the title and abstract screening, studies were included if they met all the following criteria: included suspected or confirmed sepsis, severe sepsis, or septic shock in adult patients according to the definition of Sepsis-1, 2, or 3.0(3–5); were conducted in the mainland of China and published between 1 January 1992 and 1 June 2020; reported or provided enough data to calculate; were restricted to English publications. Studies with a population of fewer than 20 people, pediatric patients, editorial or review, and geographical location not suitable were excluded.

### Data extraction and quality assessment

Three authors (Y-CL, YY, and M-MY) conducted the literature search and extracted the data. Any uncertainties with the inclusion and exclusion criteria and data extraction were discussed and consensus was reached by consensus. Variables extracted from each study were the name of the first author, the name of the study, the year of publication, the type of study, the geographical location, the number of study centers, the definition criteria of sepsis, the location of patients (ER, hospital or ICU), sample size, age, sex, the number of deaths, and the type of outcome measure (length of follow-up, 28–30 days mortality, 90 days mortality). The risk of bias was evaluated via ROBINS-I tool. Publication bias was evaluated by the Egger test and Begg funnel plot.

### Statistical analysis

Random-effects models were used to estimate pooled frequencies and mortality from sepsis, septic shock, and severe sepsis. Separate pooled random effects mortality analyzes were performed in the following subgroups: definition of sepsis-3 and definition and non-sepsis-3 definition; 28–30 days and in-hospital mortality; retrospective and prospective studies; single-center and multiple-center studies; ICU, hospital wards, and location of the ED. Statistical heterogeneity was visually assessed using forest plots and formally using the *I*^*2*^ statistic; heterogeneity was considered high for *I*^*2*^ values greater than 50%. All analyzes were performed using Stata software (StataCorp LP, College Station, TX, USA) version 14.0 and the package 'metan'.

## Results

**Fig. 1:**
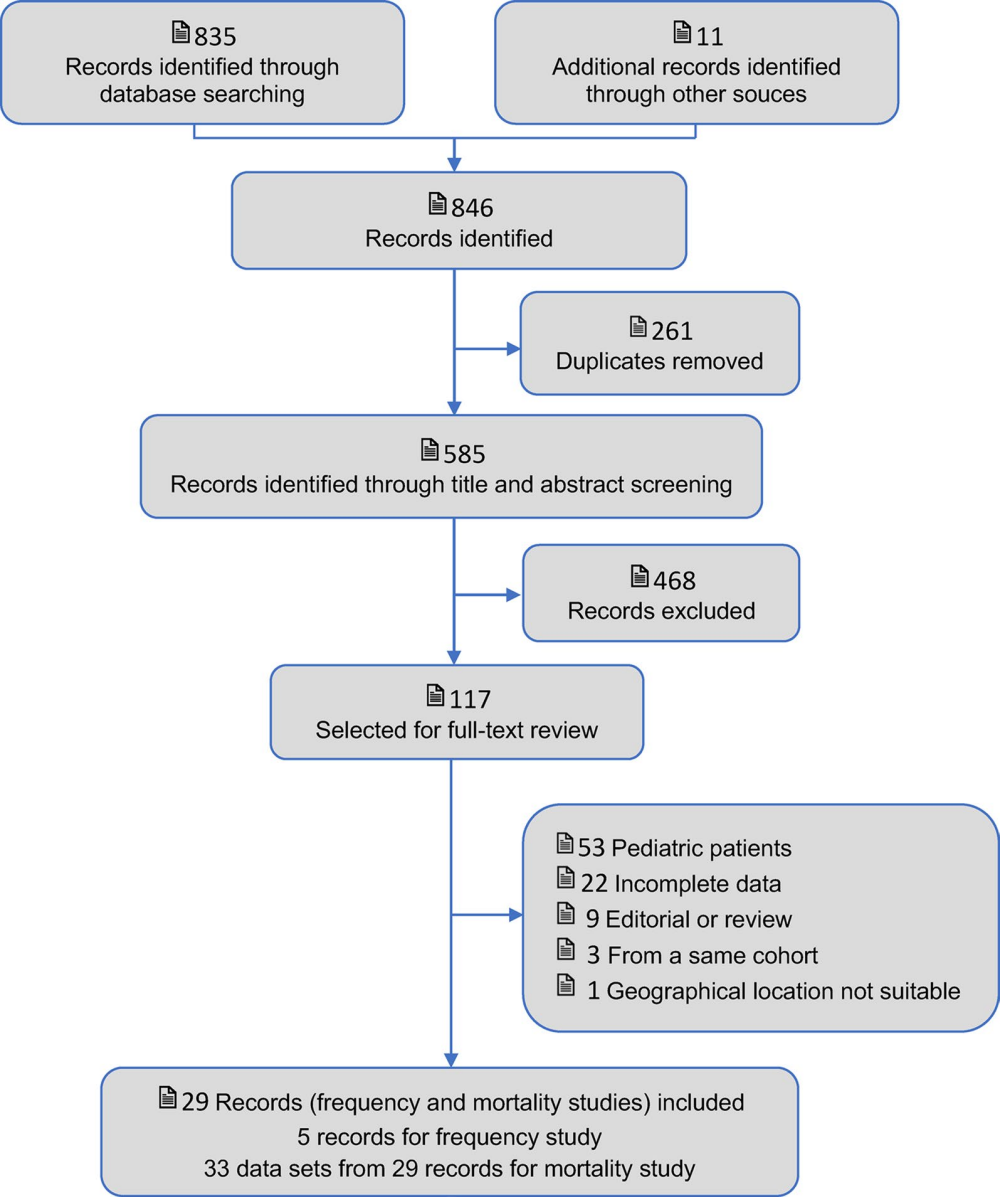
HYPERLINK "sps:id::fig1||locator::gr1||MediaObject::0" PRISMA flow chart of studies. PRISMA flowchart describing the process of selecting studies eligible for meta-analysis

The searches yielded 846 citations. After 261 duplicates were removed, we reviewed the titles and abstracts, and 468 articles were excluded. Of the remaining 117 studies, 88 were excluded after reviewing the full article. A total of five studies for frequency and 29 studies (33 data sets) for mortality were included in the review. Figure 1 shows the study flow for the selection process. Risk of bias in included studies were assessed via ROBINS-I tool (Additional file 1: Figs. S1 and S2). Funnel plots were used to describe the publication bias on sepsis and septic shock mortality (Additional file 1: Fig. S3). Meta-regression analyses on sepsis and septic shock mortality were conducted using the “metareg” function in Stata (Additional file 1).

### Descriptive characteristics

Frequency data were reported in 5 studies (7 data sets) that covered 6,852 patients (five were conducted in the ICU and two were conducted hospital-wide). Mortality data was reported in 29 studies (33 data sets), including 12,108 septic patients (sepsis, severe sepsis, and septic shock) (Table 1). Most of the included studies were carried out in the ICU (n = 23), followed by ED (n = 4) and hospital admission studies (n = 2).

**Table 1:** Summary of studies reporting the frequency and mortality of sepsis and septic shock in China

Author	Year	Study design	No. of centers	Patients Location	Diagnosis Criteria	Sepsis mortality: cases/total number screened (%)	Septic shock mortality: cases/total number screened (%)	Severe sepsis mortality: cases/total number screened (%)	Study details
Cheng [22]	2007	Prospective	10	ICU	Sepsis-1	NA	NA	142/318 (44.7) (28-day)	Epidemiology of severe sepsis in critically ill surgical patients
Yang [23]	2007	Prospective	6	ICU	Sepsis-1	NA	NA	106/240 (44.2) (28-day)	Relationship between adrenal function and prognosis in patients with severe sepsis
Xie(24)	2008	Retrospective	10	ICU	Sepsis-1	NA	155/318 (30.5) (in-hospital)	NA	Impact of invasive fungal infection on outcomes of severe sepsis
Chen [25]	2009	Retrospective	1	ED	Sepsis-1	122/327 (37.3) (28-day)	NA	NA	Prognostic value of BNP in ED sepsis patients
Li [26]	2013	Prospective	11	ICU	Sepsis-2	NA	NA	72/218 (33.0) (28-day)	Implementing surviving sepsis campaign bundles in China
Wu [27]	2013	Prospective	6	ICU	Sepsis-1	NA	NA	110/361 (30.5) (28-day)	The efficacy of thymosin alpha 1 for severe sepsis
Wang [28]	2014	Prospective	1	ICU	Sepsis-1	87/196 (44.4) (28-day)	NA	NA	Functional polymorphisms of interferon-gamma affect pneumonia-induced sepsis
Xu [29]	2014	Prospective	1	ICU	Sepsis-1	NA	9/58 (15.5) (28-day)	NA	Effect of two volume responsiveness evaluation methods on fluid resuscitation and prognosis in septic shock patients
Yin [30]	2014	Prospective	1	ED	Sepsis-2	225/680 (33.1) (28-day)	NA	NA	Thrombosis and haemostasias scoring system for overt disseminated intravascular coagulation in ED sepsis
Zhou [31]	2014	Prospective	22	ICU	Sepsis-1	NA	51/119 (42.9) [28-day]	162/484 (33.5) (28-day)	Epidemiology and Outcome of Severe Sepsis and Septic Shock in Intensive Care Units
Luo [32]	2015	Prospective	1	ICU	Sepsis-1	NA	11/29 (38.0) (28-day)	NA	Increased cardiac index attenuates septic acute kidney injury
Wu [33]	2015	Prospective	1	ICU	Sepsis-2	NA	NA	17/62 (27.4) (28-day)	Hypermetabolism is related to a poor outcome in Severe Sepsis
Li [34]	2016	Prospective	7	ICU	Sepsis-1	NA	48/199 (29.0) (28-day)	NA	Effects of Shenfu injection in the treatment of septic shock patients
Lu [35]	2016	Retrospective	1	ICU	Sepsis-2	NA	NA	18/68 (26.5) (28-day)	Peripheral T‑lymphocyte and NK cell imbalance is associated with septic encephalopathy
Cheng [36]	2017	Retrospective	6	ICU	Sepsis-1, Sepsis-3	Sepsis-1: 58/186 (31.2) (28-day) Sepsis-3: 58/175 (33.1) (28-day)	NA	NA	Comparison of the performance between sepsis-1 and sepsis-3 in ICUs in China
Wang [37]	2017	Prospective	1	ICU	Sepsis-3	NA	72/240 (30.0) [28-day]	NA	Effect of levosimendan on elderly septic shock patients
Yan [38]	2017	Prospective	1	ICU	Sepsis-2	NA	NA	30/63 (25.4) (28-day)	Prognostic value of left ventricular-arterial coupling in elderly patients with septic shock
Huang [39]	2018	Prospective	1	ED	Sepsis-1	4/39 (10.3) (28-day)	17/57 (29.8) [28-day]	8/55 (14.5) (28-day)	Lp‑PLA2 predict mortality rates of septic patients
Zhou [40]	2018	Prospective	1	ICU	Sepsis-2	74/178 (41.6) (in-hospital)	NA	NA	Impact of BMI on survival of medical patients with sepsis
Liu [41]	2018	Prospective	21	ICU	Sepsis-2	NA	205/617 (33.2) (28-day)	NA	Terlipressin versus norepinephrine as infusion in patients with septic shock
Jiang [42]	2019	Retrospective	1	Hospital-wide	Sepsis-3	19/98 (19.4) (28-day)	NA	NA	Analysis of prognostic factors of septic pulmonary embolism patients
Liang [43]	2019	Retrospective	1	ICU	Sepsis-3	NA	146/232 (62.9) (28-day)	NA	Prognostic factors of mortality in Septic patients with mechanically ventilation
Xing [44]	2019	Prospective	5	ICU	Sepsis-1	58/301 (19.3) (28-day)	NA	NA	Traditional Chinese medicine bundle therapy for septic acute gastrointestinal injury
Xue [45]	2019	Prospective	1	ICU	Sepsis-1	NA	NA	18/71 (25.4) (28-day)	Alterations of Th2/Th1 in previously immunocompetent patients with community-acquired severe sepsis
Zhang W [13]	2019	Prospective	1	ICU	Sepsis-3	188/631 (29.8) (28-day)	NA	NA	Systemic inflammatory response syndrome in Sepsis-3
Zhang Z [46]	2019	Retrospective	1	ICU	Sepsis-2	473/1997 (23.7) (in-hospital)	NA	NA	Prolonged stay in ED increased risk of hospital mortality in septic patients
Dong [14]	2020	Retrospective	111	Hospital-wide	Sepsis-1, Sepsis-3	Sepsis-1: 353/1716 (20.6) (in-hospital) Sepsis-3: 299/935 (32.0) (in-hospital)	NA	NA	External validity of Adult Sepsis Event’s simplified SOFA criteria
Xie [8]	2020	Prospective	44	ICU	Sepsis-1	824/2322 (35.5) (90-day)	NA	NA	The epidemiology of sepsis in Chinese ICUs
Zhao [47]	2020	Retrospective	2	ED	Sepsis-3	95/316 (30.1) (28-day)	NA	NA	Serum ammonia levels for predicting sepsis patient mortality

### Sepsis frequency

Five studies reported that the frequency of sepsis in the ICU ranged from 20.6% (8) to 50.8% (13). The overall pooled frequency of sepsis was estimated at 33.6% (95% CI 25.9% to 41.3%) with a high level of heterogeneity (I^2^ = 99.2%; p < 0.001). Only one study reported the sepsis frequency in hospital wards patients [14], 8.1% in the Sepsis-1 criteria, and 4.4% in Sepsis-3 criteria. There is a lack of studies on the frequency of sepsis in the ED and population-based studies. Because of the small number of included studies and the changing definition of sepsis, trends in sepsis frequency were not sufficient for analysis.

### Mortality of sepsis

**Fig. 2:**
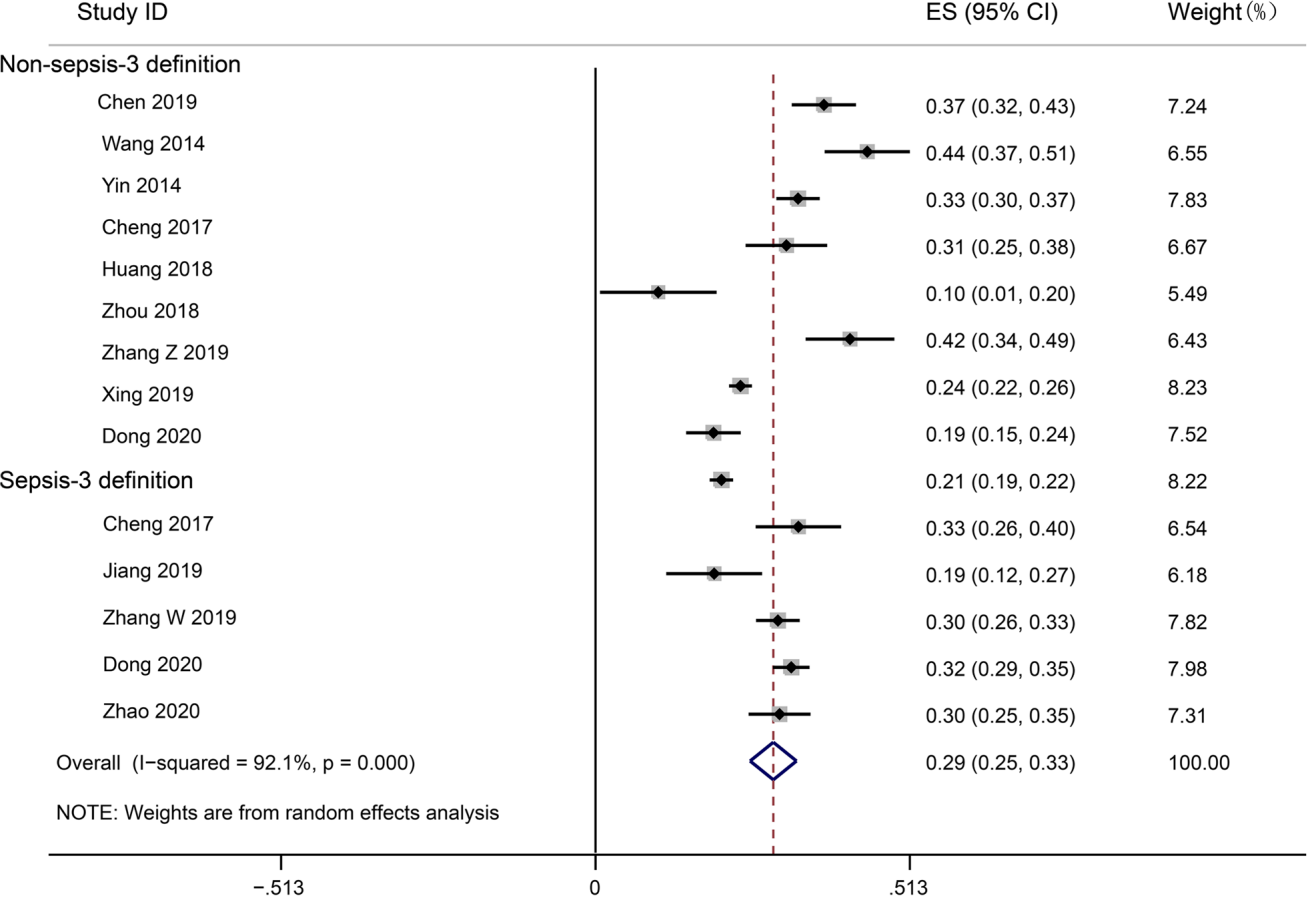
Random effects meta-analysis of studies reporting mortality of sepsis patients. The forest plots contain exact 95% confidence intervals, and specific studies are weighted using the inverse-variance method

Fourteen studies (7775 participants) reported that sepsis mortality (28-30d or in-hospital) of sepsis ranged between 10.3% and 44.4%. The pooled mortality was 29.0% (95% CI 25.3%–32.8%), and I^2^ for heterogeneity of 92.1% indicated high heterogeneity (Fig. 2). Only one study reported the mortality at 90 days mortality (2322 participants), which was 33.5%, much higher than the mortality at 28–30 days and in-hospital mortality.

**Fig. 3:**
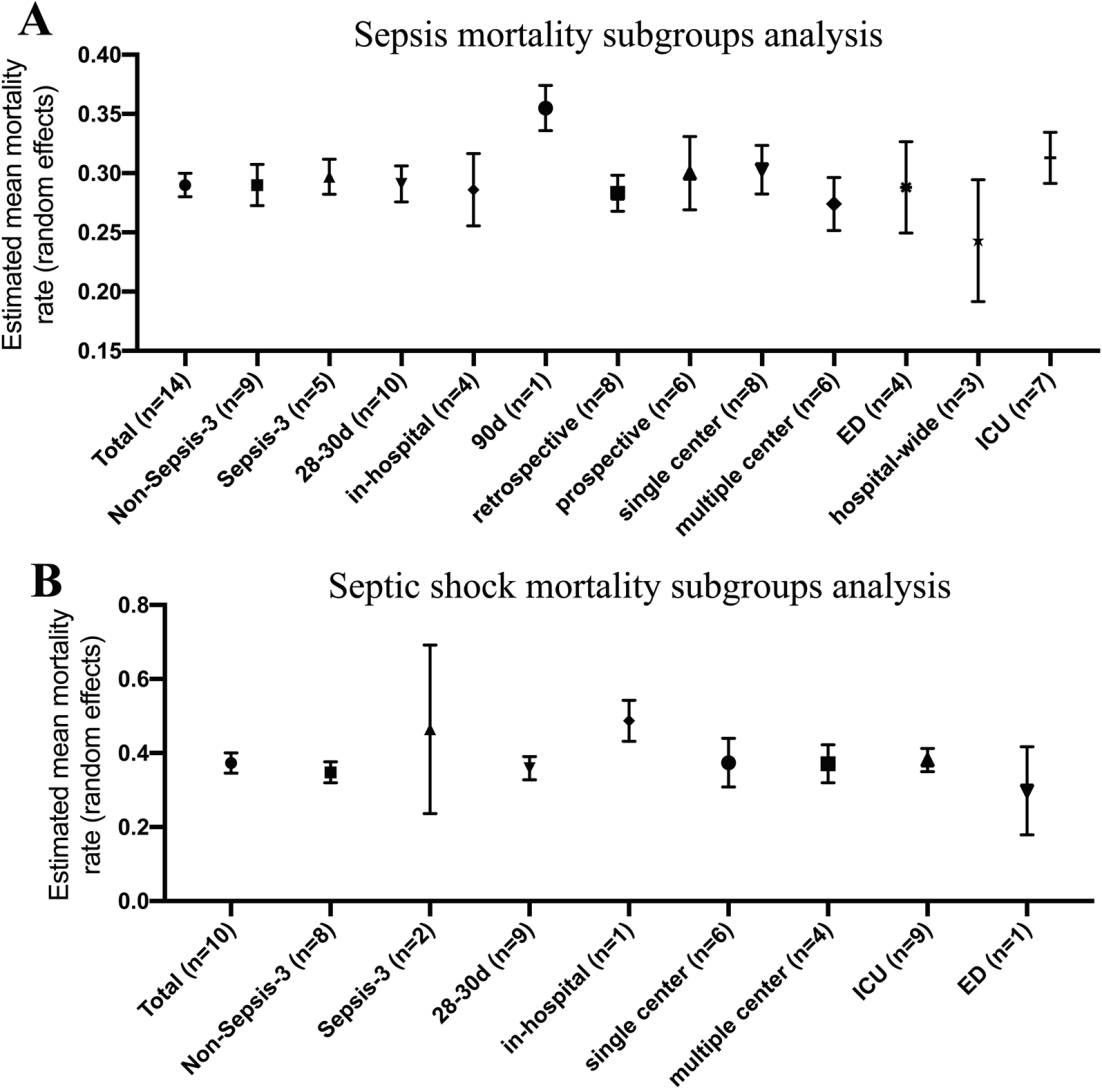
Subgroups analysis of sepsis and septic shock mortality. Comparison of pooled mortality rates of sepsis **A** and septic shock **B** derived from non-sepsis-3, sepsis-3, 28-30d, in-hospital, 90d, prospective, retrospective, single center, multiple centers, ED, hospital-wide, and ICU studies, showing rates and 95% confidence intervals

Five subgroups meta-analysis using random-effects model were performed to evaluate the mortality of sepsis (Fig. 3A). Data from the definition of sepsis-3 (N = 5 studies with 2,115 participants) reported an average mortality rate of sepsis of 29.7% (95% CI 26.4%–33.0%, I^2^ = 57.4%) compared to the definition of studies (N = 9 studies with 5,620 participants) of 29.0% (95% CI 23.8%–34.2%, I^2^ = 94.0%). Observed mortality at 28–30 days of sepsis (N = 10 studies with 2949 participants) was 29.1% (95% CI 24.3%–34.0%, I^2^ = 87.8%), while mortality (N = 4 studies with 4826 participants) was 28.6% (95% CI 22.5%–34.6%, I^2^ = 95.2%). Retrospective studies (N = 8 studies with 5750 participants) reported an average sepsis mortality rate of 28.3% (95% CI 24.0%–32.6%, I^2^ = 91.1%) compared to prospective studies (N = 6 studies with 2025 participants) of 30.0% (95% CI 22.3%–37.6%, I^2^ = 92.6%) Single center studies (N = 8 studies with 4146 participants) reported an average sepsis mortality rate of 30.3% (95% CI 24.4%–36.1%, I^2^ = 92.6%) compared to multiple center studies (N = 6 studies with 3629 participants) of 27.4% (95% CI 21.9%–33.0%, I^2^ = 91.7%). N = 7 studies with 3664 participants conducted in ICU reported an average sepsis mortality rate of 31.3% (95% CI 25.6–37.1%, I^2^ = 91.4%), compared to studies conducted in hospital wards (N = 3 studies with 2749 participants) of 24.3% (95% CI 15.4%–33.1%, I^2^ = 95.1%) and studies conducted in ED (N = 4 studies with 1362 participants) of 28.8% (95% CI 21.1–36.4%, I^2^ = 87.9%).

### Mortality of severe sepsis

**Fig. 4:**
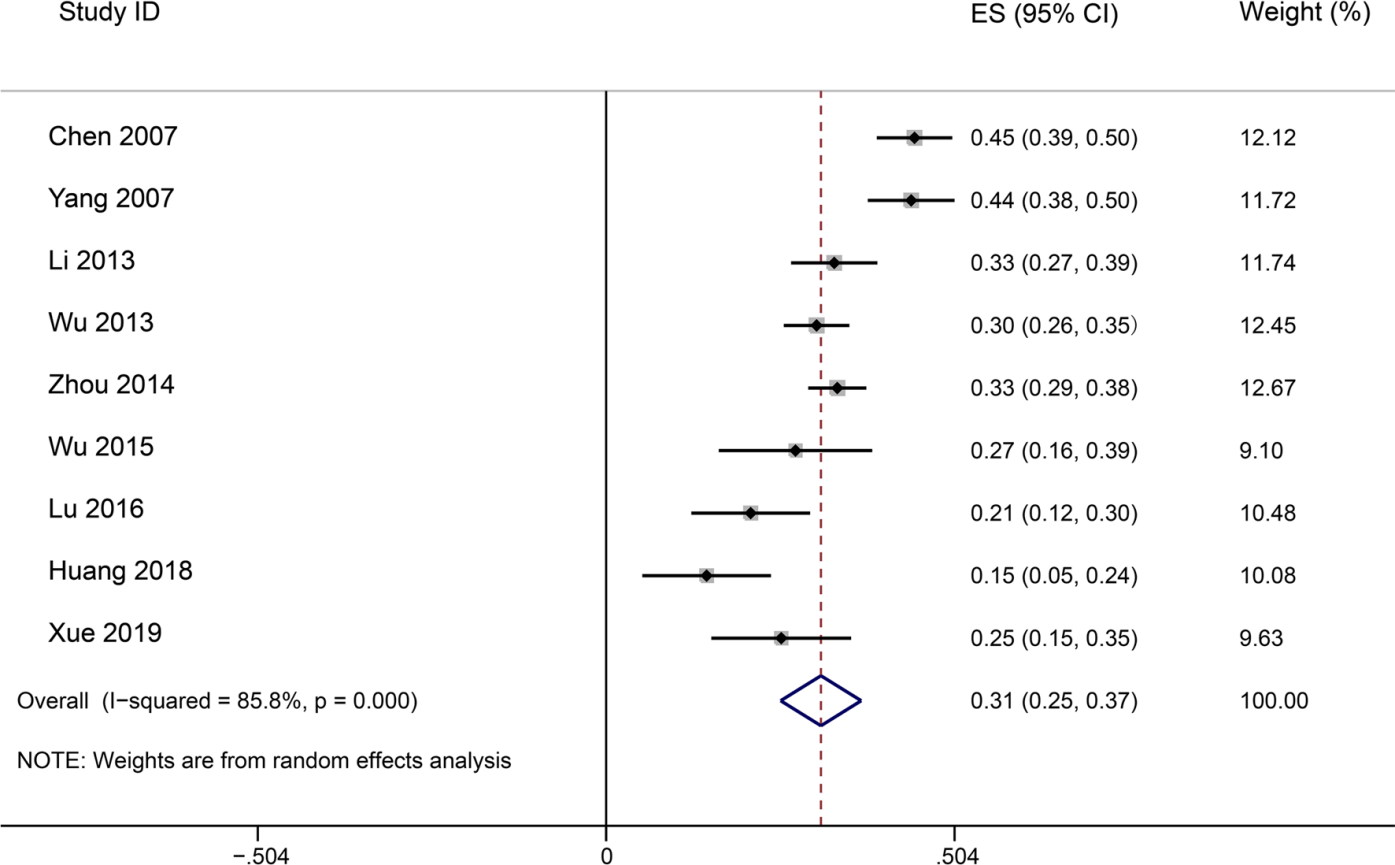
Random effects meta-analysis of studies reporting mortality of severe sepsis patients. The forest plots contain exact 95% confidence intervals, and specific studies are weighted using the inverse-variance method

The mortality rate of severe sepsis defined before Sepsis-3 was evaluated in nine studies, with 1895 participants. The overall pooled mortality of severe sepsis was estimated at 31.1% (95% CI 25.3% to 36.9%) with a high level of heterogeneity (I^2^ = 85.8%; p < 0.001) (Fig. 4).

### Mortality of septic shock

**Fig. 5:**
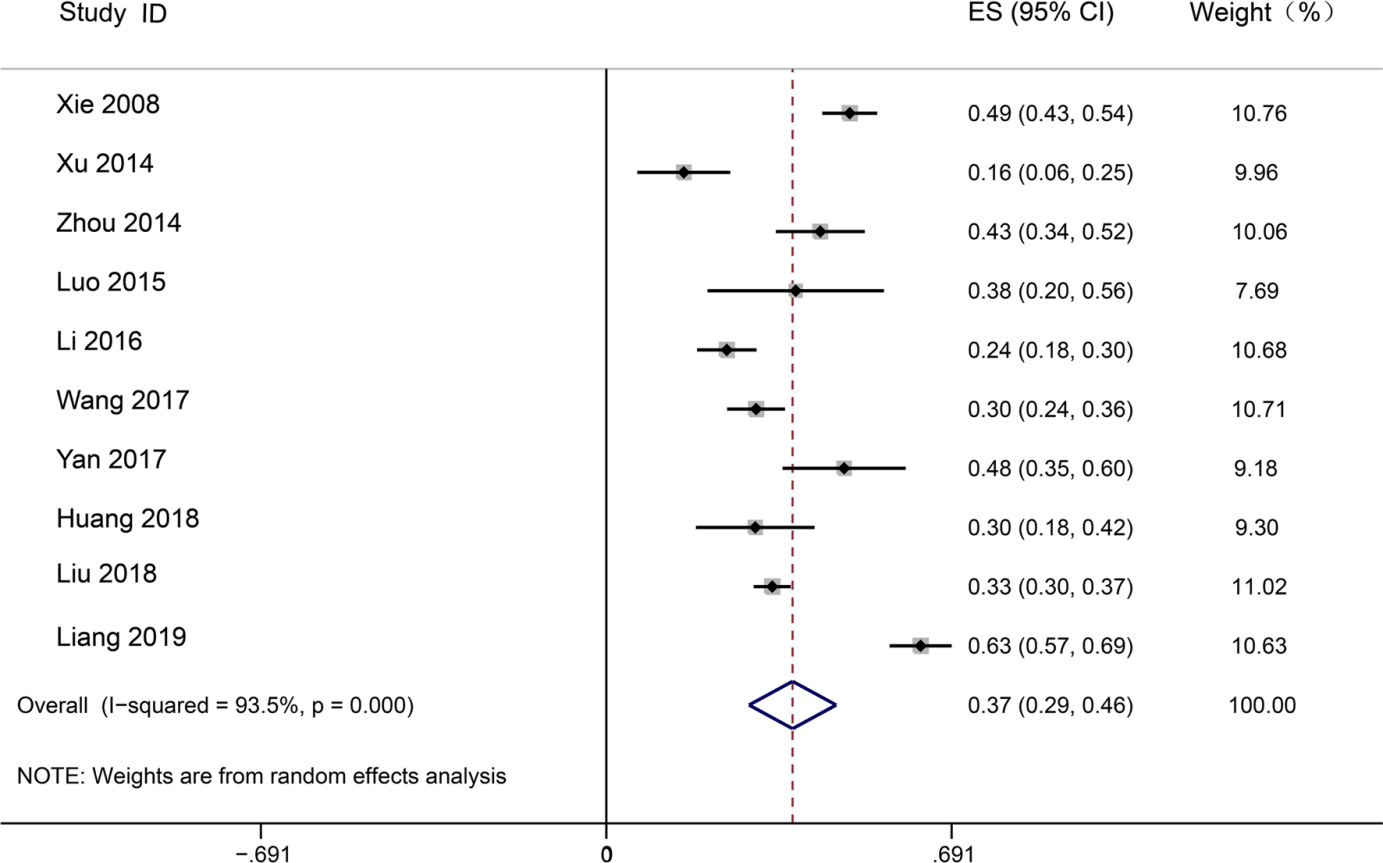
Random effects meta-analysis of studies reporting mortality of septic shock patients. The forest plots contain exact 95% confidence intervals, and specific studies are weighted using the inverse-variance method

Data on septic shock mortality (28-30d or in-hospital) were obtained from ten studies, and 1932 participants. The pooled mortality rate was 37.3% (95% CI 28.6%–46.0%), I^2^ for 93.5% heterogeneity indicated high heterogeneity (Fig. 5). Meta-analysis of four subgroups using a random-effects model was performed to evaluate the mortality of septic shock (Fig. 3B). Two studies using the sepsis-3 definition reported an average mortality rate of 46.4% (95% CI 14.2%–78.7%, I^2^ = 98.3%), whereas eight studies using the non-sepsis-3 definition reported an average mortality rate of 34.8% (95% CI 26.8%–42.8%, I^2^ = 88.8%). Single center studies (N = 6) reported an average mortality rate from septic shock of 37.4% (95% CI 21.3%–53.4%, I^2^ = 94.7%) compared to multiple center studies (N = 4) of 37.1% (95% CI 26.8%–47.4%, I^2^ = 92.6%). Mortality of septic shock at 28/30 days (N = 9) was estimated at 35.9% (95% CI 26.5% to 45.3%, I^2^ = 93.3%), while only one study was observed in-hospital mortality, which was 48.7%, much higher than 28/30 days. Nine studies carried out in the ICU reported an average mortality rate from septic shock of 38.1% (95% CI 28.8–47.4%), while only one study carried out in the ED reported a mortality rate of 29.8%.

## Discussion

To our knowledge, this systematic review is the first to investigate sepsis epidemiology in the mainland of China. Our result shows that 33.6% of patients in ICU have a diagnosis of sepsis on the mainland of China. The overall mortality of pooled sepsis (28-30d or in-hospital) is 29.0% (95% CI 25.3%–32.8%) with high heterogeneity. The overall mortality (28-30d or in-hospital) in septic shock and severe sepsis are 37.3% (95% CI 28.6%–46.0%) and 31.1% (95% CI 25.3% to 36.9%). Most of the data were extracted from ICU studies and a huge demand for the epidemiology of sepsis in the ED, hospital ward, or population-based studies are urgently needed.

Bauer et al. [6] and Vincent et al. [7] reviewed the frequency and mortality of sepsis and septic shock specifically in Europe and North America, however, the epidemiology of sepsis and septic shock in Asia, Africa, and South America countries were excluded due to non-comparable healthcare systems [15]. Studies revealed that the frequency of septic shock in Europe and North America was 10.4% at ICU admission and 8.3% at any time during the ICU stay. The frequency of sepsis diagnosed at any time of ICU stay was estimated at 34% in China, while there is not enough data on the frequency of septic shock in hospital wards, ED, or population-based studies. Our results show that the average mortality of 30-day sepsis in China is 29.5%, which is higher than 24.4% in Europe and North America. The overall mortality from septic shock in China is 37.3%, which is also higher than 33.7% in North America, 32.5% in Europe, and 26.4% in Australia.

Several reasons may explain this difference. First, with unevenly distributed medical resources, ED crowding is especially serious in China [16], which is a major barrier for septic patients to receive timely emergency care. Second, there are only 3.6 ICU beds per 100,000 capita in China compared with 34.7 in the US and 29.2 in Germany [17]. Third, the residency and fellowship training program for critical care medicine in China was not established until April 2020. Clinicians have deficiencies in understanding the concept evolution and the standard treatment of sepsis, especially the non-emergency and ICU clinicians. Fourth, China's population is severely aging [18], which also increases the frequency and mortality of sepsis mortality, as the incidence of sepsis is disproportionately increased in elderly adults, and age is an independent predictor of mortality [19]. Finally, antimicrobial resistance caused by misuse and overuse of antibiotics also increases the mortality of sepsis [20].

Based on our findings, we offer the following recommendations for healthcare systems to improve the frequency and mortality of sepsis in China. Strengthening the construction of an emergency triage system and improving the current situation of ED crowding. Increasing the per capita ratio of beds in the ED and ICU beds. Strengthening the critical care medicine professional training of non-emergency and ICU clinicians. Standardizing the rational use of antibiotics.

Our study has some limitations. First, there is a lack of studies on the frequency of sepsis, especially after the definition of Sepsis-3. Several studies reported the frequency of sepsis in the ICU; however, there are very few studies in hospital wards and ED. Prompt identification and appropriate treatment of sepsis in the ED are crucial to improving patient outcomes [21], therefore, studies on the frequency of sepsis in the ED are desperately needed in future research on sepsis. Second, we pooled the 28-30d mortality and in-hospital mortality together in the sepsis and septic shock mortality research due to the insufficient number of studies, which may have an impact on the results. Third, the heterogeneous inclusion criteria of included studies induced by the evolving definition of sepsis may also have an impact on the combined results. Fourth, unbalanced regional development in China led to a lack of data in economically underdeveloped regions, which may result in low estimates of the frequency and mortality of sepsis and septic shock.

Our study has some strengths. To our knowledge, this is the first meta-analysis of epidemiological studies of Chinese sepsis. We included not only observational studies but also interventional or RCT studies. This broad inclusion criteria represent a more realistic range of patients with sepsis and septic shock.

## Conclusions

Our study provides the first systematic review to investigate the epidemiology of sepsis on China's mainland. Our findings indicated that the frequency and mortality of sepsis and septic shock in China were much higher than North America and Europe countries. Based on our results, an extremely high incidence and mortality of sepsis and septic shock in China's mainland require more healthcare budget support. Epidemiological data on sepsis and septic shock in the ED are severely lacking; More research is urgently needed in this area.

## Supplementary Information

**Additional file 1.** Meta-regression and Additional figures.

## Data Availability

The data sets used during the current study are available from the corresponding author on a reasonable request.
