# Supplementary material for: Frequency and mortality of sepsis and septic shock in China: a systematic review and meta-analysis
Source: BMC Infect Dis. 2022 Jun 21;22:564. doi: 10.1186/s12879-022-07543-8 (PMC9210671; doi:10.1186/s12879-022-07543-8)

Additional file 1: Meta-regression and Additional figures.

Meta-regression

1. Sepsis mortality

Meta-regression	Number of obs = 14
REML estimate of between-study variance	tau2 = .0113
% residual variation due to heterogeneity	I-squared_res = 91.99%
Proportion of between-study variance explained	Adj R-squared = -61.65%
Joint test for all covariates	Model F(5,8) = 0.13
With Knapp-Hartung modification	Prob > F = 0.9825

_ES	Coef.	Std. Err.	t	P>|t|	[95% Conf. Interval]	
Definition	.019325	.0350875	0.55	0.597	-.0615869	.1002369
Study design	.0098267	.0719412	0.14	0.895	-.15607	.1757235
Study location	-.0290154	.0558661	-0.52	0.618	-.1578428	.099812
Endpoints	.0310449	.0814382	0.38	0.713	-.156752	.2188418
Study centers	-.0185944	.0701453	-0.27	0.798	-.1803497	.1431608
_cons	.2844725	.2191471	1.30	0.230	-.2208817	.7898267

2. Septic shock mortality

Meta-regression	Number of obs = 10
REML estimate of between-study variance	tau2 = .01358
% residual variation due to heterogeneity	I-squared_res = 87.42%
Proportion of between-study variance explained	Adj R-squared = 24.45%
Joint test for all covariates	Model F(4,5) = 1.53
With Knapp-Hartung modification	Prob > F = 0.3220

_ES	Coef.	Std. Err.	t	P>|t|	[95% Conf. Interval]	
Definition	.0245329	.1276626	0.19	0.855	-.3036343	.3527
Study design	-.2249434	.1081076	-2.08	0.092	-.5028429	.052956
Study location	-.0291704	.1532626	-0.19	0.857	-.4231445	.3648036
Study centers	.0133103	.1025027	0.13	0.902	-.2501813	.2768019
_cons	.7553199	.3030357	2.49	0.055	-.0236582	1.534298

Figure S1

Risk of bias domains

Study	D1	D2	D3	D4	D5	D6	D7	Overall
Chen 2009	-	X	?	?	-	+	X	X
Wang 2014	+	X	?	?	+	+	-	-
Yin 2014	+	-	?	?	+	-	-	-
Cheng 2017b	+	X	?	?	+	-	+	-
Huang 2018	!	X	?	?	-	-	-	X
Zhou 2018	X	X	?	?	+	+	-	-
Zhang Z 2019	+	+	?	?	+	+	-	+
Xing 2019	-	+	+	-	+	+	-	+
Dong 2020	+	+	?	?	+	+	+	+
Xie 2020	+	+	?	?	+	+	+	+
Jiang 2019	+	-	?	?	+	+	-	-
Zhang W 2019	+	+	?	?	+	-	-	-
Zhao 2020	X	!	?	?	-	-	X	X
Cheng 2017a	+	+	?	?	+	+	+	+
Yang 2007	-	-	?	+	+	+	-	-
Li 2013	-	+	?	?	-	+	-	+
Wu 2013	+	-	+	-	+	+	-	+
Zhou 2014	+	+	?	?	-	+	-	-
Wu 2015	!	X	-	?	X	-	X	X
Lu 2016	X	X	?	?	-	-	!	X
Xue 2019	-	X	?	?	-	-	X	-
Xie 2008	+	+	?	?	+	+	-	+
Xu 2014	-	+	+	+	-	+	-	+
Luo 2015	+	X	-	-	-	+	X	-
Li 2016	-	+	+	-	+	+	-	+
Wang 2017	X	-	-	-	+	+	X	-
Yan 2017	+	-	?	?	-	+	-	-
Liu 2018	+	+	+	+	+	+	+	+
Liang 2019	!	X	?	?	-	-	X	X

Domains:
D1: Bias due to confounding.
D2: Bias due to selection of participants.
D3: Bias in classification of interventions.
D4: Bias due to deviations from intended interventions.
D5: Bias due to missing data.
D6: Bias in measurement of outcomes.
D7: Bias in selection of the reported result.

Judgement
! Critical
X Serious
- Moderate
+ Low
? No information

Figure S2

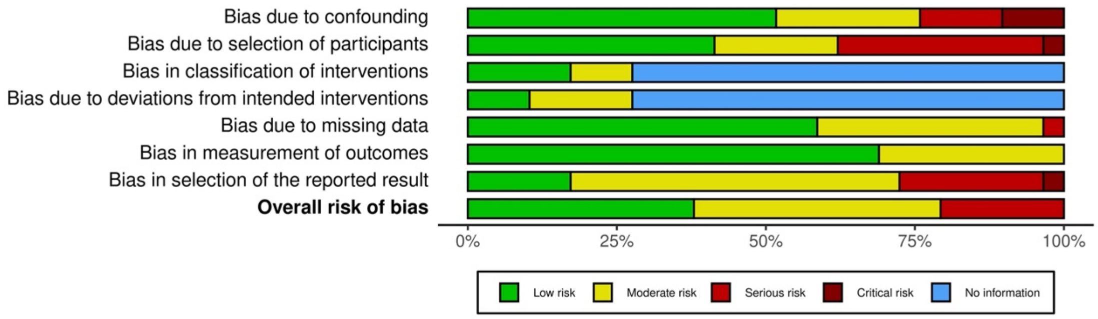

Figure S3

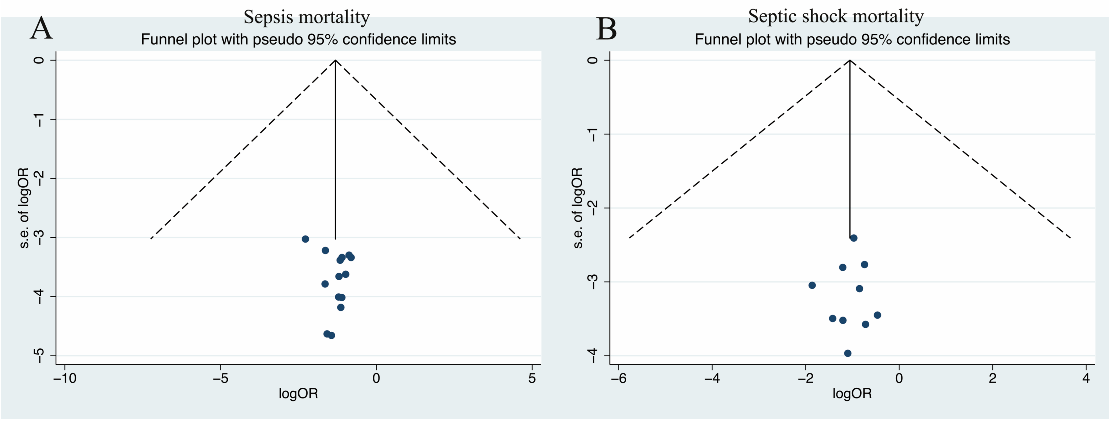

Supplement: Supplementary file 1 — Additional file 1. Meta-regression and Additional figures. [file 12879_2022_7543_MOESM1_ESM.pdf]
